# Symbolic Play among Children with Autism Spectrum Disorder: A Scoping Review

**DOI:** 10.3390/children8090801

**Published:** 2021-09-12

**Authors:** Francisco González-Sala, Irene Gómez-Marí, Raúl Tárraga-Mínguez, Alba Vicente-Carvajal, Gemma Pastor-Cerezuela

**Affiliations:** 1 Department of Developmental and Educational Psychology, University of Valencia, 46010 València, Spain; alvicar2@alumni.uv.es; 2 Department of Education and School Management, University of Valencia, 46010 València, Spain; 3 Department of Basic Psychology, University of Valencia, 46010 València, Spain; gemma.pastor@uv.es

**Keywords:** autism spectrum disorder, neurodevelopmental disorders, pretend play, symbolic play, toddlers

## Abstract

Symbolic play is considered an early indicator in the diagnosis of autism spectrum disorder (ASD) and its assessment. The objective of this study was to analyze the difficulties in symbolic play experienced by children with ASD and to determine the existence of differences in symbolic play among children with ASD, children with other neurodevelopmental disorders and children with typical development. A scoping review was carried out in the Web of Science (WoS), Scopus, ERIC, and PsycInfo databases, following the extension for scoping reviews of the Preferred Reporting Items for Systematic Reviews and Meta-Analyses (PRISMA) statement. The number of papers included in the review was 22. The results confirm that children with ASD have greater difficulties with symbolic play than children with other neurodevelopmental disorders and children with typical development, even when controlling for their verbal age. Difficulties are greater in situations of free or spontaneous play. Results evidenced that the absence or deficiency in the symbolic play can serve as an early indicator of ASD between the first and second year of life, the developmental moment in which this type of play begins.

## 1. Introduction

### 1.1. Neurodevelopmental Disorders: Autism Spectrum Disorder

The last classification of the Diagnostic and Statistical Manual of Mental Disorders (DSM-5) concerning neurodevelopmental disorders include a wide range of neurological based disorders that have their origin in childhood, affect child development itself, and are characterized by deficits in different areas, such as the personal, social, academic, and occupational [1]. Some of the diagnoses included in this categorization are as follows: autism spectrum disorder (ASD), intellectual disability (ID), communication disorders, specific learning disorders, or attention deficit hyperactivity disorder, among others.

Due to the great heterogeneity in the presentation of the clinical forms of neurodevelopmental disorders and the variability of areas that can be affected, clinical diagnosis could be hindered. In the case of ASD, it is a neurodevelopmental disorder characterized by persistent difficulties in communication and social interaction in different contexts, and by reproducing repetitive and restricted patterns of behaviors, activities, and interests. These symptoms appear in the early stages of development and are not better explained by intellectual disability (intellectual developmental disorder) or global developmental delay. These disturbances are permanent and have an impact in affective, academic, occupational and social areas, among others, causing a clinically significant deterioration [1]. Emotional, communicative and symbolic development can be, in fact, affected in people with ASD [2].

### 1.2. Symbolic Play Development in Childhood

Play has an important role in children’s development [3,4], providing motor, cognitive and, mainly, the social skills [5,6] related to symbolic play [7,8,9]. Some studies [10,11,12,13,14] describe a common pattern in the development of the game, starting with manipulative and exploratory plays, followed by functional and, ultimately, symbolic plays.

Symbolic or simulation play arises between 18 and 24 months of life [9,15]. It is characterized by the use of objects with a different role to their specific one, that is, assigning features to objects that they do not present, as a consequence of the use of imagination [16,17]. In this kind of play, children acquire different learnings, imitating situations that happen mainly in their day to day lives [18,19,20]. For example, the typical scenario of using a block of building games as if it were a car [18], phoning with a banana [19], holding a bottle using the hand of a doll [20], or turning their own body into a plane. Leslie [21] distinguishes three kinds of symbolic play depending on the moment of appearance: the use of objects as if they were others, the attribution of false features to an object, and, finally, referring to an object as if it was present. The classification of Sigman and Ungerer [22] includes manipulative play, relational play, functional play, and symbolic play, considering the last one as the most complex.

### 1.3. Autism and Symbolic Play

In the case of children with ASD, one of the most common features is the difficulty they usually experience regarding symbolic play [23,24,25,26]. These difficulties are even considered an early indicator for the diagnosis of autism [23] and its assessment [18]. This is also reflected in the DSM-5 [1] by including, among the symptoms of ASD, behaviors such as the repetition of a particular kind of game, the stereotyped use of objects, the alignment of toys, the presence of very restricted interests, and deficiencies related to the imagination.

According to some studies [24,27], in the first year of life, different play patterns can be detected in children with developmental disorders when comparing to children of typical development. In the case of children with ASD, symbolic play lacks diversity and complexity, instead being repetitive and limited [28]; it is not spontaneous (as if it were a type of learned play [29]) rare and varied [25,26].

Baron-Cohen and Howlin [30] indicate that difficulties in playing in children with ASD are due to cognitive problems, such as the fact of understanding the mental states of others. Along this line, Leslie [21] points to the difficulty of children with ASD in mentally associating two representations, one concerning the real world and the other to pretend to be another identity. This second representation, associated with ToM, has been pointed out in children with typical development to pretend play, by Taylor and Carlson [31] and Suddendorf et al., [32]. According to Baron-Cohen [20], children with ASD have a representation of the world as it is, and not as it is not. Bigham [33] points out that the more different are the referent and the substitute (that is, the representation of the real world and the representation feigned), the more difficult it is to relate both kinds of representations for the child with ASD.

However, Harris [34] relates the difficulties that children with ASD have in “pretend play” when it comes to their limited knowledge of the real world. That makes it difficult to act as if something were something else.

Another theory to explain the difficulties of children with ASD in this type of game has been the weak central coherence theory [35,36]. This points out that children with ASD have a strong preference to process the information given in a local context versus a more global context. This circumstance makes it difficult for the child to understand and perform certain acts (not real, fake, nonliteral) in a specific context, such as a play context.

Bigham [37] points out the greater predictive value of mental abilities in the difficulties in the generation of pretend play compared to other theories, such as that of the local processing of information and response inhibition theory.

Riviere [38] and Mundy and Sigman [39] relate the social, communicative, and language limitations of people with ASD to the development of symbolic and fictional capacities. These difficulties in symbolic play can be shaped so that children with ASD can substitute one object for another [40,41], although they are not novel games and lack spontaneity.

Research shows the existence of a remarkable relationship among child play, the age of development [42], and the development of expressive and receptive language [41,43,44,45,46,47] in children with both typical and atypical development. According to Bigham [37], the delay in the development of language and receptive language predicts the difficulties of children with ASD in pretend play. This is related to the hypothesis of symbolic deficit proposed by Baron-Cohen [20] and Ricks and Wing [48]. They argue that the difficulties in symbolization are linked to deficits in language, simulation, and mentalization.

This kind of play in children with ASD has been related to certain factors, such as type of attachment, mental age, mental abilities, and verbal competence [49,50,51,52,53]. Thus, children with secure attachment present a higher level of symbolic play and spend more time playing than children with disorganized attachment [49]. Symbolic play has also been associated with theory of mind (ToM) [50,51] and verbal skills [52]. In this sense, a study by Chang et al. [53] found a relationship between a greater presence of symbolic play and a higher level of expressive language in children with ASD. Some authors [54] considered it necessary, in the study of symbolic play, to match children with ASD with children of typical development according to their language development level. Finally, the absence of symbolic play in children with ASD does not mean that they cannot learn it. Intraverbal training, that is, telling a child that a particular object is another (for example, stating that a plate is a hat), leads the child to use a plate as a hat, increasing substitution symbolic play situations [55,56].

Thus, taking all this information into account, the objective of this study was to synthesize, through a scoping review, the conclusions of research conducted in recent decades about the difficulties in symbolic play experienced by children with ASD and to analyze the possible implications for the early identification and diagnosis of ASD. We structured the scoping review according to one main and three secondary questions.

The initial question that guided this review was as follows:

- Are the difficulties of children with ASD in symbolic play generalizable to all the papers, carried out in recent years, included in this scoping review?
- From this main question, three secondary questions subsequently arose:
- Are these difficulties different from those experienced by children with other neurodevelopmental disorders and/or children with typical development?
- Can the situation of play, spontaneous play or by imitation, influence the greater or lesser presence of symbolic play?
- Is there any relationship between the variable ‘verbal age of the children’ and symbolic play?

## 2. Methods

### 2.1. Search Strategy

This scoping review was carried out following the extension for scoping reviews of the “Preferred Reporting Items for Systematic Reviews and Meta-Analyses” (PRISMA) [57] statement (for further details, see Supplementary Table S1).

The databases consulted for the bibliographic search were Web of Science (WoS), Scopus, ERIC, and PsycInfo. The initial search was conducted on 23 December 2020, combining the terms “Autism”, “Asperger”, “ASD”, “Symbolic Play”, “Symbolic Game”, “Pretend Play”, “Typical Development”, “Development Delay” through the Boolean operators “AND” and “OR”. The combinations of terms were as follows:

- (autism* OR Asperger OR ASD) AND (“symbolic play” OR “symbolic game” OR “pretend play”) AND (“typical development” OR “development delay”) in the field of topic in the case of WoS.
- (autism* OR Asperger OR ASD) AND (“symbolic play” OR “symbolic game” OR “pretend play”) AND (“typical development” OR “development delay”) in the fields of title, abstract and keywords, in the case of Scopus.
- (autism* OR Asperger OR ASD) AND (“symbolic play” OR “symbolic game” OR “pretend play”) AND (“typical development” OR “development delay”) in the field of title and abstract, in the case of ERIC.
- (autism* OR Asperger OR ASD) AND (“symbolic play” OR “symbolic game” OR “pretend play”) AND (“typical development” OR “development delay”) in anywhere, in the case of PsycInfo.

The period delineated for the search was from 1943, the year in which the first scientific article on autism was published [58], to 2020.

### 2.2. Selection Criteria

The inclusion criteria were: (a) papers written in English or Spanish; (b) that compare symbolic play in children with ASD, with typical development and/or with other neurodevelopmental disorders; (c) with a chronological age or a verbal mental age not older than 6 years.

Furthermore, descriptive and/or theoretical papers that did not provide empirical data about the topic of the review were excluded; papers that did not include a comparison group in addition to the group of children with ASD were also excluded; and research not published in scientific journal papers (such as books and conference proceedings) was also excluded.

### 2.3. Study Selection

As a result, a total of 81 papers were obtained: 50 in ERIC, 17 in WoS, 11 in PsycInfo, 2 in Scopus and 1 from another source.

The references of each of the selected papers were reviewed, performing a reverse search process as indicated by Urrutia and Bonfill [59], to assess useful references that had not appeared in the initial search, adding, by that means, a doctoral thesis. Finally, the search was repeated in the same conditions at the end of January 2021, to find any possible article published at the end of 2020. However, no new study was found.

Of the 81 selected papers, after the first screening, 30 were eliminated because they were duplicated in at least two databases. Ten more studies were also removed because they were not scientific papers.

The title and abstract of the remaining 41 papers were reviewed. After that, 8 studies that did not include a comparison group and 4 studies not related to symbolic play were removed.

After this, the remaining 29 papers were downloaded and fully reviewed. Seven of them were excluded because they did not match with the object of the present study. Thus, the final number of papers selected for the review was 22.

This whole process of analysis to determine the suitability, or not, of the papers to the objective of the review was made by two of the authors. Both of them, independently, applied the inclusion and exclusion criteria. After independent reviews, cases in which there was divergence in data collection, a consensus was reached because of the participation of a third researcher.

**Figure 1:**
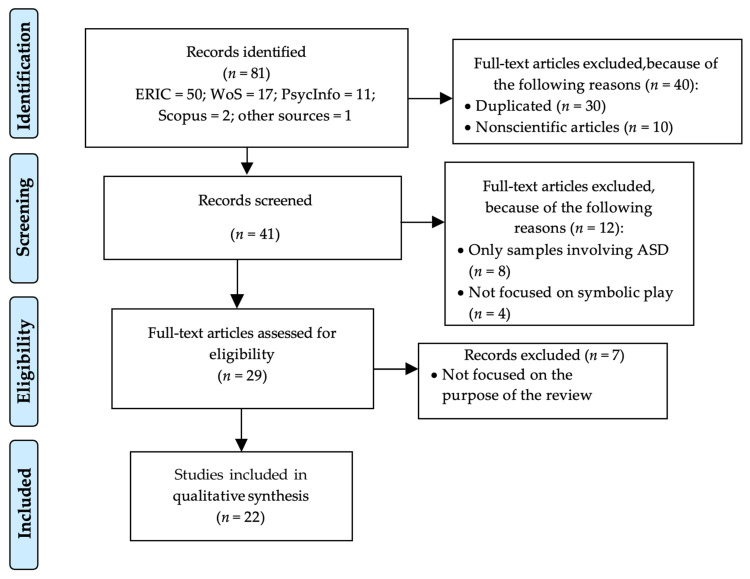
Flow diagram (according to PRISMA) used in this scoping review. Abbreviations included in the figure: ASD: Autism Spectrum Disorder; ERIC: Education Resources Information Center; WoS: Web of Science.

Regarding the methodological quality of the papers selected for this scoping review, this was assessed through 10 out of the 18 indicators included in the SQUIRE Guidelines 2.0 quality scale [60]. These indicators were title, abstract, problem description, specific aims, measures, analysis, results, limitations, and conclusions. Two researchers applied the quality criteria. The papers were classified into three categories according to their quality: low, medium, and high; and only those classified in the high category were included in the review. None of the selected papers were eliminated. The whole process is shown in Figure 1.

### 2.4. Data Extraction

The information concerning each of the selected studies was as follows: authors who signed the article; year of publication and journal; the objective of the study; groups of participants and sample size; and descriptive characteristics of the samples, such as chronological age, mental age, and nonverbal mental age, among others.

## 3. Results

Table 1 shows a summary of the main characteristics of the 22 papers analyzed in this review, including the following information: study and year of publication; the purpose of each study; characteristics of the sample according to the number of children, groups included in the studies, and chronological, verbal and nonverbal age (when specified in the study); play related study variables; and main results.

### 3.1. Characteristics of the Participating Samples

A total of 4 out of 22 papers included in this review compared the symbolic play of children with autism and children with other neurodevelopmental disorders [18,49,67,69]; five studies compared children with ASD with children with typical development [15,17,51,52,65]; and thirteen papers included children with ASD, children with other diagnoses and typical development children [20,22,28,33,42,50,61,62,63,64,66,68,70].

Of the papers specifying the type of neurodevelopmental disorders, six of the papers had a group with Down syndrome [20,28,42,61,63,64]; one paper included a group with hearing impaired children, another included a language impaired group, another a group with an intellectual disability [62]; and another paper included a group with a moderate degree of a specific learning disorder [33].

The sample sizes range from 24 [51] to 130 children [70]. Table 2 shows the ranges of chronological, verbal and nonverbal mental age of the samples included in the studies. As shown in Table 2, higher chronological age ranges correspond to children with ASD and other developmental disorders, while the verbal mental age ranges are lower, to be able to equalize the children of the different groups in verbal age [54]. Specifically, only 11 papers match the verbal age of children with ASD with the children of the other study groups [20,33,51,52,61,63,64,66,67,69,70].

The samples included in the studies of the present review have been equalized in some variables, such as chronological age, [15,18,20,49,50,51,52,65,66,67,68,69,70] and mental, verbal or nonverbal age, such as [15,17,18,22,28,33,42,51,61,63,64,66,67,69,70]. In the case of the study by Rutherford and Rogers [50], groups are equalized in mental age and in nonverbal mental age, while there do exist differences in verbal mental age or QI [15,18,52]. Other papers show differences in chronological age [17,22,33,42,61,66] or in mental age [49] among the involved groups.

### 3.2. Instruments Used for the Assessment of the Symbolic Play

Regarding the assessment of the symbolic play, eight studies used a standardized instrument. Specifically, two of them [50,66] used the Fewell Play Scale, 5th edition [71], which consists of introducing the child to toys and waiting for him/her to play spontaneously so that, if he/she does not, some behavior is modeled by the experimenter. The test ends when the child performs three consecutive symbolic play actions or three consecutive errors. Two other papers [15,18] used an adapted version of the Developmental Play Assessment (DPA [72,73]), which allows the evaluation of the play in unstructured and semi-structured tasks for 15 min. The examiner models a symbolic play-act using each imaginary object, except for free play, where he/she can only touch and imitate the actions and toys the child performs.

Two other studies [69,70] used The Test of Pretend Play (ToPP [74]), which covers three aspects of the symbolic play: the ability to substitute one object for another, to refer to an object that is not present, and to attribute an absent property to an object. Those aspects are carried out in a semistructured task, where the examiner provides the child opportunities to imitate what he/she has modeled. However, not all the meanings of symbolic toys are shaped, so that they can also be generated spontaneously. In the study of [28], the Child-Initiated Pretend Play Assessment (ChIPPA) [75] was used during a 30 min session. In the first five minutes, the child is encouraged to play with toys alone. In the second five minutes, the examiner models five symbolic play actions, and in the last minutes, does not model any action. Finally, Pierce [68] uses the Communication and Symbolic Behavior Scales Developmental Profile (CSBS [76]), which allows the child to play spontaneously before modeling some actions. Thus, play is only modeled to encourage the child.

In other papers, the symbolic play was classified according to categories that had been established in previous studies. Thus, Bentenuto et al. [42] encoded the symbolic play into four categories, as had been carried out in other studies [77,78]. They differentiated self-directed pretense, other directed pretense, sequential pretense, and substitution pretense. Some studies [17,49] used the symbolic play categories of object substitution, adding a pretend property to a toy, pretending that something exists when it does not or role-playing [21]. Other studies [64,65] used the categories listed in the classification of Libby et al. [64]. Specifically, these categories of symbolic play are object substitution, the attribution of false properties, and reference to an absent object. There are also other categories, such as: not attending, attending, not acting, unrelated behavior, labeling, showing, attempting to terminate the session, exploration, sensorimotor play, relational play, and functional play.

### 3.3. Differences in Symbolic Play between Children with ASD and Children with Other Neurodevelopmental Disorders

Regarding symbolic or simulation play comparison between children with ASD and children with other neurodevelopmental disorders, some studies [20,22,50,61,66,67,68,69,70] found that children with ASD present less symbolic play than children with other neurodevelopmental disorders. The study by Bigham [33] found greater difficulties in children with ASD compared to children with typical development and children with moderate learning disabilities in simulation play, highlighting that these difficulties are greater when the referent is less related to the substitute, either by its form or by its function, suggesting that greater decontextualization impairs the understanding of simulation play in children with ASD.

In contrast, some studies [18,42,49] did not find differences between the groups of children with ASD and other neurodevelopmental disorders. On the one hand, [63,64] did not find differences either, when the proposed tasks of play to the child are simple; on the other hand, in the face of more complex tasks, children with ASD have worse outcomes than children with Down syndrome. A study by Lee et al. [28] did not find differences between children with ASD, with Down syndrome, and with typical development when it comes to playing by imitation; however, there are differences between the group with ASD and the other two groups when the game is free, with the group with ASD being the one that presents greater limitations in the production of symbolic play. Stone et al. [62] did not find differences in the number of symbolic play behaviors among children with ASD, children with neurodevelopmental disorders, and children of typical development, although the number of children with ASD performing a symbolic and functional play is lower.

### 3.4. Differences in Symbolic Play between Children with ASD and Children with Typical Development

When the comparison is made between children with ASD and of typical development, it is found in most studies that children with ASD present greater difficulties in symbolic play [15,17,20,28,50,51,61,66,67,70]. In other cases, there were no differences between the two groups [42,62,65]. In some of the studies that show differences between the two groups, it was evident that children with ASD present half of the symbolic play actions than children with typical development [15,20,50,66]. Finally, in the study of Libby et al. [63], no differences were found between the two groups in simple symbolic play tasks, but in complex play tasks; while in the study of Warreyn et al. [52], they found differences, but not related to symbolic play by imitation.

In short, and taking into consideration the differences in symbolic play between children with ASD and the two other groups considered, those with ASD spent less time in symbolic states of play than the rest [70], showed less elaborate simulation games [24], and also had a poorer fluidity and symbolic content of the simulation play [51,61]. Likewise, when the verbal ages between groups are equalized, all the studies concluded a lower symbolic game in the group of children with ASD than in the other groups [20,51,52,61,63,64,66,67,69,70].

### 3.5. Differences in Symbolic Play Modeling (or by Imitation) and Free Play (or Spontaneous) between Children with ASD, Other Neurodevelopmental Disorders and Typical Development

In the case of free and spontaneous play, children with ASD presented fewer symbolic or simulation play actions than children with typical development [15,17,20,50,51,52,66] or with other neurodevelopmental disorders [20,50,66]. However, in other studies, these differences were not obtained [42,65]. In the study by Libby et al. [64], differences were only obtained in situations that implied the absence of an object or the attribution of false properties to objects. However, there were no differences among children with ASD, Down syndrome, and typical development concerning the substitution set. Regarding the study by Naber et al. [49], although there was no difference between spontaneous play and play by imitation, the situation of play exposed to children was spontaneous play, since the mother did not intervene unless the child requested it. In this study, no differences were found concerning manipulative, functional and symbolic play, between children with ASD and children with other neurodevelopmental.

Finally, seven papers have studied symbolic modeled play in children with ASD. Four of them [28,61,67,68] found that, although children with autism presented simulation play less frequently than other groups, in the case of structured, guided and modeled tasks, the difficulties of children with ASD significantly decreased. Even Warreyn et al. [52] found no differences between children with ASD and typical development in symbolic play by imitation. This type of symbolic play (modeling or by imitation) gives children with ASD a higher level of play than free play [61]. Similar results are found in the study by Hobson et al. [67], in which it is observed that children with ASD benefit considerably from modeling play. Likewise, Lee and other researchers [28] found that children with autism present difficulties in the generation of symbolic play, but not in its imitation. However, it is important to note that modeled symbolic play significantly improves the symbolic abilities of children with ASD. This play is less frequent than that of children with other neurodevelopmental disorders and with typical development [61]. However, two papers [63,64] obtain better results for the ASD group than for the typical developmental and Down syndrome groups in terms of simple symbolic play imitation, although these differences are not statistically significant.

## 4. Discussion

The purpose of this study was to synthesize, through a scoping review, the conclusions of research conducted in recent decades on the difficulties in symbolic play experienced by children with ASD and to analyze the possible implications for the early identification and diagnosis of ASD.

Of the 22 studies analyzed, only three of them found no differences between the group involving children with ASD and the group with another neurodevelopmental disorder [18,42,62]; and four studies did not find differences between the group involving children with ASD and the group involving children with typical development [42,49,62,67]. In the case of the study by Bentenuto et al. [42], it is necessary to take into account the age of the children, which is about 21 months, being below 24 months, the age from which the difficulties in symbolic play in children with ASD are more evident [52]. Along the same line, Thiemann-Bourque et al. [18] pointed out that the absence of differences between children with ASD and other disorders may be related to the lack of language.

Two papers did not find differences between the group with children with ASD and the group with neurodevelopment disorders in terms of simple symbolic play, in which they have to imitate a single action, with differences evident when they have to imitate a symbolic play with different actions [63,64]. However, 15 studies did find differences between symbolic or simulation play between children with ASD and children with other neurodevelopmental disorders, or typical development [15,17,20,22,28,33,50,51,52,61,66,67,68,69,70]. All these data help us to answer the initial and main question that structures this article, since we could confirm that children with ASD have greater difficulties in the production of symbolic play than children with other neurodevelopmental disorders and children with typical development.

According to Lam and Yeung [51], the difficulties in symbolic play or simulation in children with ASD can be explained by the theory of mind. To this end, difficulties in understanding another person’s mind are significantly related to the shortcomings of the simulation play. This idea is consistent with Leslie [21], who pointed out that children need to have two representations in mind simultaneously to produce symbolic play. In this sense, the study by Rutherford et al. [66] highlighted the importance of joint attention to the capacity of metarepresentation, understanding it as a forerunner to the development of the theory of mind and, thus, a predictive variable of symbolic play in the three groups examined.

Likewise, some authors also highlighted, as a predictive variable of the simulation play, mental age, since they hypothesize the possibility that this game develops as an individual matures cognitively. Moreover, a study by Bigham [33] pointed out the difficulties of children with ASD to understand the simulation play, while two studies [54,79] did not find differences in understanding this type of games between children with ASD and typical development. However, Bigham [33] contended that the greater the decontextualization between the referent and what is simulated, the more difficulties in understanding there are. Thus, responding to our second question, the differences are significantly higher in the group of children with ASD, since there are some inherent characteristics of ASD that make it difficult for children with this disorder to produce the symbolic play, such as deficiencies in metarepresentation skills related to ToM [21], generating ideas [40] or the lack of joint attention abilities [44,80,81,82].

Children with ASD also have a lower level of symbolic play than other groups in free play situations, as obtained by [15,17,20,50,51,52,66]. Therefore, the conclusions drawn by several studies allow us to respond to the third of our questions: children with ASD have greater difficulties in the production of spontaneous or free symbolic play than the children in the other groups. This can even be related to some aspects of executive functioning, such as rigidity and poor cognitive flexibility, which would make it difficult to attribute an object a quality or a not properly attributable use.

In addition, and also regarding the third question, the group of children with ASD presents greater difficulties than the group of children with neurodevelopmental disorders and typical development in the production of the symbolic play by imitation or modeling.

However, children with ASD benefit to a greater extent in the production of symbolic play in modeled play situations than in free play situations [28,52,61,63,64,67,68]. This is because children with ASD present difficulties in generating games, but not always with the imitation of them, so the difficulty would be associated with a lack of imagination [28]. This fact coincides with previous studies that showed that children with ASD present difficulties in free symbolic play [24], implying that they have significant limitations in their spontaneous production abilities [83]. To this end, it is fairly clear that the fact that children with ASD present higher levels of play in modeled situations than in spontaneous situations [83] is because they imitate symbolic activity, without performing an interpretation of it, in a guided context [63]. According to a study by Lee et al. [55], children with ASD who can verbalize, are between 3 and 6 years and with the presence of functional play and imitation, can develop a substitution of symbolic play with objects through training.

Finally, studies in which children have been equalized according to their verbal age [20,51,52,61,63,64,66,67,69,70] also concluded that the group involving children with ASD presents a lower symbolic play than children with other neurodevelopmental disorders and children with typical development. This conclusion addresses our fourth and last question, related to knowing whether having the same verbal mental age provides the same levels of symbolic play in children with ASD than in other children. We state that the group of children with ASD show greater difficulties than children with other neurodevelopmental disorders and children with typical development, although they are equalized in verbal mental age. In addition to the role that language plays in the development of symbolic play [18,52,84,85], as was met in the answer to our second question, some inherent characteristics of the disorder make it difficult for children with ASD to produce symbolic games.

An important limitation of some of the included studies is the small (lower than 50 participants) sample sizes [15,17,20,22,49,51,52,61,63,67,68]. Another limitation is that not all the included studies used a symbolic game evaluation tool; and the context in which the analyzed studies were carried out: in a laboratory setting, structured and with specific toys, and not a natural one [15,18,52,67,68]. This can condition the amount and type of symbolic play presented by the children included in the studies. Concerning the selection process of the reviewed papers, the limitations are related to the number of databases conducted (not including, for instance, the Scielo database), and to the type of document (excluding Library of Congress, books, and chapters of books).

Future research should address the study of symbolic play in children with ASD, taking into account issues such as the degree of autism, more natural and closer settings to the child, and using children’s peers as models. In addition, studies are required to assess the impact of learning symbolic play by modeling on the different areas of a child’s development. In this sense, it might be considered whether this learning has an effect on the development of language, and whether this type of learned game can be generalized to other toys and contexts.

## 5. Conclusions

The results of this scoping review allow us to confirm that the absence or deficiency in symbolic play, mainly in situations of spontaneous play, can serve as an early indicator of ASD diagnosis between the first and the second year of life, just at the developmental moment at which symbolic play begins. These difficulties occur in children with ASD with and without verbal language. However, in children with ASD with verbal language, it may be more unnoticed because of the relationship between language development and play. In children with ASD, symbolic or simulation play is less frequent, less complex, and differs significantly from symbolic play skills than in children with other neurodevelopmental disorders and children with typical development. In addition, children with ASD benefit from imitation or modeled play situations. That indicates some reliance on the model when carrying out this type of game. In these cases, participation in symbolic play increases, as well as in more drawn-up simulation game proposals.

While the lack of symbolic play in children with ASD is a characteristic considered for the assessment of the disorder [18,23], this scoping review highlights the importance of assessing symbolic play, especially in spontaneous and natural play situations (and not in modeled situations). That differentiation should be considered both in clinical observation situations and in family interviews.

Eventually, the conclusions in this scoping review invite the exploration of the potential benefits of including specific interventions to promote the appearance of symbolic play, both in prevention programs for children at risk of having ASD, and in intervention programs aimed at language development for reducing social and communication difficulties of children with ASD.

## Figures and Tables

**Table 1:** Main characteristics of the studies analyzed in the scoping review.

Study	Object	Sample	Study Variables	Main Results
Riguet et al. (1981) [61]	To assess the ability of children with ASD for symbolic play in modelled situations and in free play	*n* = 30: 10 ASD, 10 DS, 10 TD. MCA (years): ASD (10.0), DS (9.5), TD (2.9). VMA 2.5 years	Free and modeled symbolic play.	The symbolic fluidity and content of symbolic play of children with ASD are poor compared to other children with a similar mental age. Symbolic play is less frequent in children with ASD. Modeling provides a higher level of play in children with ASD, but lower than in other children.
Sigman & Ungerer (1984) [22]	To compare sensorimotor skills and play behaviors among children with TD, ID, and ASD.	*n* = 48: 16 TD, 16 ID, 16 ASD CA (months): TD (16–25), ID (32–80), ASD (39–74). Developmental age (months): ID (17–38), ASD (18–38)	Free functional and symbolic play. Symbolic play after receiving an order. Sensorimotor abilities.	There are no differences in sensorimotor abilities between groups. The group of children with ASD presents less diversity in functional play and less free symbolic play, after receiving indications. There is a relationship between receptive language and functional and symbolic play in the three groups.
Baron-Cohen (1987) [20]	To evaluate the simulation play of children with ASD, comparing it with that of children with SD and with TD.	*n* = 30: 10 ASD, 10 DS, 10 TD. MCA (years): ASD (8.0), DS (7.5), TD (4.1). VMA: ASD/DS (2.5 years). N-VMA: ASD (4.9 years), DS (3.8 years)	Free simulation play.	The group of children with ASD present less spontaneous simulation play. Children with ASD who present symbolic play differ significantly in terms of the verbal and nonverbal mental age of those children with ASD who did not present symbolic play.
Stone et al. (1990) [62]	To determine whether play and motor imitation skills are different among children with ASD, children with ID, and children with communication disorders	*n* = 91: 22 ASD, 15 HI, 19 LI, 15 ID, 20 TD MCA: ASD (4.6), HI (4.2), LI (4.5), ID (5.2) NH (4.3). IQ: ASD (54.1), HI (109.8), LI (97.6), ID (56.9), NH (100). Verbal communication: ASD (3.6), HI (2.6), LI (2.2), ID (1.9) NH (1.2)	Free play. Imitation play. Game type (simple, relational, functional, symbolic). Number of game acts. Play time.	The group of children with ASD uses fewer toys than the LI, HI, and NH children’s groups, and has less playtime and appropriate playtime. Children with ASD present less functional play, with no differences concerning manipulative, relational and symbolic play. The number of children with functional and symbolic play is lower in the group of children with ASD. They have fewer imitation skills.
Libby et al. (1997) [63]	To determine the existence of differences between children with ASD, DS and TD in the imitation of simulation games of a single action and different actions.	*n* = 30: 10 ASD, 10 DS, 10 TD. MCA (months): ASD (120), DS (55), TD (28). Equalized in comprehension and expression from 30 to 32 months.	Simulation play. Task type: simple or multi-scheme (follow guidelines). Attitudes.	The group of children with ASD presents greater imitation responses in simple tasks than the TD group. In multischeme tasks there are no significant differences between the three groups, presenting the group of children with ASD with worse scores. In imitation tasks, the DSA group presents a more participatory attitude than children with TD and DS.
Libby et al. (1998) [64]	To determine the existence of differences in spontaneous play between children with ASD, Down syndrome and typical development with a verbal age of approximately 2 years.	*n* = 27: 9 ASD, 9 DS, 9 TD. MCA (years): ASD (10.03), DS (4.04), TD (2.01) Equalized in comprehension and expression from 26 to 29 months	Object search, play with objects and simulation play.	There are no differences in exploration time. The group of children with ASD presents more sensorimotor play and less relational play. There are no differences in functional play with conventional objects between groups. The group of children with ASD presents less symbolic play than the other groups. There are no differences between the groups in symbolic play where an object is substituted for another, but there are differences in symbolic play concerning the absence of an object or attributing a false feature to the object.
Rutherford & Rogers (2003) [50]	To compare the simulation play between children with ASD, DD and TD using the cognitive fundamentals of the simulation play (ToM and EF).	*n* = 78: 28 ASD, 24 DD, 26 TD. MCA (months): ASD (33.93), DD (34.83), TD (19.46). VMA (months): ASD (13.36), DD (21.42), TD (23.92). N-VMA (months): ASD (24.88), DD (23.41), TD (22.52)	Simulation play Predictive variables: ToM and EF.	Both spontaneous and induced simulation play in children with ASD are lower than in children with other disorders and with typical development. A relationship between ToM and the symbolic play of children with ASD is established, but not with executive functions. There is no significant correlation between verbal mental age and simulation play in children with ASD.
Warreyn (2005) [52]	To compare spontaneous symbolic play, imitation of symbolic play, declarative joint care and social reference between children with ASD and the control group.	*n* = 40: 20 ASD, 20 Control group. MCA (months): ASD (58.70), Control (62.25). IQ: ASD (68.80), Control (70.70) Language age (months): ASD (38.17), Control (47.17)	Symbolic play, social referencing, joint attention and symbolic imitation.	The group of children with ASD gets less symbolic and nonfunctional play and less joint care with their mothers. However, there are no differences in social reference and symbolic play by imitation, compared to the control group.
Dominguez et al. (2006) [65]	To compare the characteristics of spontaneous play among children with ASD and TD matched in chronological age.	*n* = 58: 24 ASD, 34 TD. MCA (months): ASD (36.96) TD (56.32).	Symbolic play. Playing time. Preference for toys	There are no differences in symbolic and functional play between children with ASD and TD. There are differences in sensorimotor and relational play. Children with ASD have greater play.
Rutherford et al. (2007) [66]	To examine simulation play problems among children with ASD, DD and TD by comparing sensorimotor development using a longitudinal design	*n* = 63: 28 ASD, 18 DD, 27 TD. MCA (months) Moment 1: ASD (33.9), DD (34.8), TD (19.5). Moment 2: ASD (57.6) DD (59), TD (30.1). VMA (months) Moment 1: ASD (16.50), DD (20.36), TD (23.83). Moment 2: ASD (29.21), DD (34.36), TD (39.5). N-VMA (months) Moment 1: ASD (23.7), DD (23.41), TD (23.41). Moment 2: ASD (38.21), DD (36.36), TD (35.9)	Spontaneous simulation play and modeling. Predictive variables: Imitation, Executive Performance, Joint Attention, Cognitive maturity.	There are significant differences in simulation play between children with ASD and the rest of the groups, both in spontaneous and modeled conditions, at the first and second times. Specifically, the group of children with ASD shows fewer examples of symbolic play than the rest of the groups. It is observed that, while children with ASD present difficulties both in the play of sensorimotor and in the play of imitation at the first moment, at the second, these difficulties only occur in the imitation play. Joint attention predicts equally symbolic play at the first and the second moment in the three groups.
Naber et al. (2008) [49]	To determine the existence of differences in manipulative, functional and symbolic play in children under 36 months with and without ASD	*n* = 41: 23 ASD, 18 DD y LD. MCA (months): ASD (29.04), DD (27.06) Mean development level (months): ASD (60.83), DD (67.78).	Game type (manipulative, functional, symbolic). Game level. Playing time. Types of toys. Attachment	There are no differences between children with and without ASD in the time spent playing or in the type and level of play. Children with ASD spend less time reading a book or playing with daily utensils or puzzles. The type of attachment has an effect on the kind, level, and time of play in children with ASD.
Bigham (2008) [33]	To determine the level of understanding of functional and simulation play in children with ASD, TD and LD.	*n* = 128: 36 ASD, 37 LD, 55 TD. MCA (months): ASD (92.56); LD (116.57); TD (92.56). VMA: ASD (57.33); LD (60.03); TD (58.56)	Functional play. Level of decontextualization of the object. Body parts as objects and gestures to represent the imaginary object.	There are no differences in functional play between ASD and TD groups. Children with ASD present more difficulties in simulation plays (substitution of an object) and simulation gestures of the object. They have more difficulty in simulation when the substitute used in the simulation is less related to the referent regarding its shape and function.
Hobson et al. (2009) [67]	To compare the symbolic play of children with ASD and children with DD by assessing self-awareness, the potential for flexibility in play and investing in symbolic meanings and creativity	*n* = 32: 16 ASD, 16 DD. MCA: ASD (9.6 years), DD/LD (10.4 years). VMA (years): ASD (5.3), DD/LD (5.0)	Symbolic play. Self-awareness, investment in symbolic meanings, creativity and fun.	Children with ASD show less symbolic play than other groups. This correlates with the lack of the qualities of symbolic play presented by children with ASD. It is observed that the modeled symbolic play benefits children with ASD.
Pierce (2009) [68]	To examine group differences in symbolic play behaviors among children with ASD, DD, and TD.	*n* = 121: 48 ASD, 25 DD, 48 TD. CA from 18 to 24 months	Symbolic, exploratory and functional play. Communicative and Symbolic Behavior Scale (CSBS).	The group of children with ASD shows less symbolic and functional play behaviors than the TD group. In contrast, concerning to exploratory play, children with ASD have a greater number of behaviors related to exploratory play than children with TD.
Lam & Yeung (2012) [51]	To compare symbolic play between children with ASD and TD. To examine which variables correlate with the symbolic play.	*n* = 24: 12 ASD, 12 TD. MCA (years): ASD (6.11), TD (5.64). VMA (months): ASD (70.17) and TD (77.91). N-VMA (months): ASD (22.83), TD (24.08)	Symbolic play. ToM: Executive operation.	The group involving children with ASD shows significant deficiencies in symbolic play concerning to the TD group. These deficiencies correlate significantly with the ToM, but not with the EF.
Thiemann –Bourque et al. (2012) [18]	To examine the differences or similarities of symbolic play between children with ASD and DD. To analyze the relationship between symbolic play and language.	*n* = 73: 35 ASD, 38 DD. MCA (months): ASD (49.2), DD (49.7).	Symbolic play: expressive communication and listening comprehension.	Similarities are obtained in the symbolic play, in terms of appearance and interest in toys, between the two groups. The diversity of symbolic play of children with ASD is similar to that of children with other DD. Significant correlations are obtained between symbolic play, language, and cognitive abilities.
Hobson et al. (2013) [69]	To examine the differences in symbolic play between children with ASD and DD. To explore the correlation of symbolic play with language, communication and social interaction.	*n* = 57: 41 ASD, 16 DD. CA 5 years. VMA: 2.9 years	Simulation play. Communication and social interaction.	Children with ASD get lower scores in symbolic play than children with other DD, even though they are matched in verbal ability. Deficiencies in communication and social interaction correlate significantly with symbolic play in children with ASD.
Strid et al. (2013) [17]	To examine how children with ASD differ from children with TD in symbolic play, deferred imitation, and type of parent comments (synchronized/nonsynchronized) during play.	*n* = 43: 20 ASD, 23 TD. MCA (months): ASD (66.8), TD (35). The group involving children with ASD was separated into two groups: mental verbal age (38.3) and non-verbal mental age (13.6).	Symbolic play, deferred imitation and parental feedback.	Both symbolic play and deferred imitation are lower in children with ASD than in children with TD. However, symbolic play is still lower in children with nonspeaking ASD. Deferred imitation and simulation play do not correlate in any of the groups.
Hobson et al. (2015) [70]	To explore the relationship between simulation play and communicative engagement in children with ASD, DD and TD.	*n* = 130: 41 ASD, 17 DD, 72 TD. MCA (years): ASD (5.7), DD (5.5), TD (3.9). VMA: ASD (2.8 years), DD (2.9 years)	Symbolic play. Communicative commitment.	Children with ASD spend less time in symbolic play states. Children with DD spend half their time in symbolic play states. Children with ASD get a lower rate of symbolic change of meaning per minute than those from the DD group, getting this rate very similar to that of children with TD.
Bentenuto et al. (2016) [42]	To analyze the characteristics of symbolic and exploratory play in children with ASD, SD and TD, through the collaborative play of mothers and children.	*n* = 75 Children and mothers: 25 ASD, 25 DS, 25 TD. MCA (months): ASD (43.33), DS (36.68), TD (20.01). Mental age (months): ASD (24.21), DS (21.12), TD (20.01)	Symbolic play, exploratory play, collaborative play of mothers with children.	There are no differences in symbolic play between the three groups, with children with ASD showing the same ability as children with DS and TD. However, children with ASD participate more in exploratory play than the other two groups.
Lee et al. (2016) [28]	To explore the relationship between simulation play and the inner experience of children’s playfulness with ASD, DD and TD.	*n* = 60: 20 ASD, 20 DS, 20 TD MCA (months): ASD (67.3), DD (71.2), TD (58.9)	Symbolic play. Playfulness.	Children with ASD show elaborate simulation plays. There is a significant relationship between simulation play and playfulness in children with ASD, so the more elaborate the play, the more joyful the experiences. Children with ASD have more difficulty generating symbolic play ideas, but not imitating them.
Thiemann -Bourque (2019) [15]	To compare symbolic and functional play skills between children with ASD and TD.	*n* = 38: 19 ASD, 19: TD MCA (months): ASD (74) and TD (34)	Symbolic play. Expressive communication and listening comprehension	Children with ASD have fewer symbolic play actions than children with TD. There are no differences between groups in functional play.

ASD = autism spectrum disorder; CA = chronological age; DD = other developmental disorders; DS = Down syndrome; EF = executive functions; HI = hearing impaired; ID = intellectual disability; LD = specific learning disorder; LI = language impaired; MCA = mean chronological age; N-VMA = non-verbal mental age; TD = typical development; ToM = theory of mind; VMA = verbal mental age.

**Table 2:** Mean age ranges (chronological, verbal and nonverbal) of the study.

Age Ranges	ASD	DD	TD
Chronological age range	1.5 to 10.3	1.5 to 10.4	1.6 to 5.6
Verbal mental age range	1.1 to 5.8	1.6 to 5	1.4 to 6.4
Nonverbal mental age range	1.9 to 5	1.9 to 5.6	1.8 to 2.9

ASD = autism spectrum disorder; DD = other developmental disorders; TD = typical development.

## Data Availability

Not applicable.

## Supplementary Materials

The following are available online at https://www.mdpi.com/article/10.3390/children8090801/s1, Table S1: Preferred Reporting Items for Systematic reviews and Meta-Analyses extension for Scoping Reviews (PRISMA-ScR) Checklist.

## References

[1] American Psychiatric Association (2013). Diagnostic and Statistical Manual of Mental Disorders.

[2] González-Moreno C.X. (2018). Intervención en un niño con autismo mediante el juego. Rev. Fac. Med. Univ. Nac. Colomb..

[3] Boucher J. (1999). Pretend play as improvisation: Conversation in the preschool classroom. Br. J. Dev. Psychol..

[4] Parham L.D., Fazio L.S. (2008). Play in Occupational Therapy for Children.

[5] Ginsburg K.R. (2007). The importance of play in promoting healthy child development and maintaining strong parent-child bonds. Am. Acad. Pediatr..

[6] Lantz J. (2001). Play time: An examination of play intervention strategies for children with autism spectrum disorders. Reporter.

[7] Betherton I. (1984). Symbolic Play: The Development of Social Understanding.

[8] Venuti P., de Falco S., Giusti Z., Bornstein M.H. (2008). Play and emotional availability in young children with down syndrome. Infant. Ment. Health J..

[9] Lillard A., Lerner M., Hopkins E., Dore R., Smith E., Palmquist C. (2013). The impact of pretend play on children’s development: A review of the evidence. Psychol. Bull..

[10] Sinclair H. (1970). The transition from sensory-motor behavior to symbolic activity. Interchange.

[11] Inhelder B., Liezine I., Sinclair I., Stambak M. (1972). Les débuts de la fonction symbolique. Arch. Psychol..

[12] Lowe M. (1975). Trends in the development of representational play in infants from one to three years. J. Child. Psychol. Psychiat..

[13] Fenson L., Kagan J., Kearsley R., Zelazo P. (1976). The developmental progression of manipulative play in the first two years. Child. Develpm..

[14] Largo R., Howard J. (1979). Developmental progression in play behavior of children between nine and thirty months. Develpm. Med. Child. Neurol..

[15] Thiemann-Bourque K., Johnson L.K., Brady N.C. (2019). Similarities in Functional Play and Differences in Symbolic Play of Children With Autism Spectrum Disorder. Am. J. Intellect. Dev. Disabil..

[16] Russ S.W. (2016). Pretend Play: Antecedent of Adult Creativity. New Dir. Child. Adolesc. Dev..

[17] Strid K., Heimann M., Tjus T. (2013). Pretend play, deferred imitation and parent-child interaction in speaking and non-speaking children with autism. Scand. J. Psychol..

[18] Thiemann-Bourque K., Brady N., Fleming K. (2012). Symbolic Play of Preschoolers with Severe Communication Impairments with Autism and Other Developmental Delays: More Similarities than Differences. J. Autism Dev. Disord..

[19] Weisberg D. (2015). Pretend play. Wiley Interdiscip. Rev..

[20] Baron-Cohen S. (1987). Autism and symbolic play. Br. J. Dev. Psychol..

[21] Leslie A.M. (1987). Pretense and Representation: The Origins of ‘Theory of Mind’. Psychol. Rev..

[22] Sigman M., Ungerer J.A. (1984). Cognitive and language skills in autistic, mentally retarded, and normal children. Dev. Psychol..

[23] Wetherby A., Woods J., Allen L., Cleary J., Dickinson H., Lord C. (2004). Early Indicators of Autism Spectrum Disorders in the Second Year of Life. J. Autism Dev. Disord.

[24] Ungerer J.A., Sigman M. (1981). Symbolic Play and Language Comprehension in Autistic Children. J. Am. Acad. Child. Adolesc. Psychiatry.

[25] Barton E.E. (2015). Teaching Generalized Pretend Play and Related Behaviors to Young Children with Disabilities. Except. Child..

[26] Desha L., Ziviani J., Rodger S. (2003). Play preferences and behaviour of preschool children with autistic spectrum disorder in a clinical environment. Phys. Occup. Ther. Pediatr..

[27] Van Berckelaer-Onnes I.A. (2003). Promoting Early Play. Autism.

[28] Lee Y., Chan P., Lin S., Chen C., Huang C., Chen K. (2016). Correlation patterns between pretend play and playfulness in children with autism spectrum disorder, developmental delay, and typical development. Res. Autism Spectr. Disord..

[29] Williams E., Reddy V., Costall A. (2001). Taking a Closer Look at Functional Play in Children with Autism. J. Autism Dev. Disord..

[30] Baron-Cohen S., Howlin P. (1993). El déficit de la Teoría de la Mente en Autismo: Algunas cuestiones para la enseñanza y el diagnóstico. Siglo Cero.

[31] Taylor M., Carlson S.M. (1997). The relation between individual differences in fantasy and theory of mind. Child. Dev..

[32] Suddendorf T., Fletcher-Flinn C., Johnston L. (1999). (1999). Pantomime and theory of mind. J. Genet. Psychol.

[33] Bigham S. (2008). Comprehension of pretense in children with autism. Br. J. Dev. Psychol..

[34] Harris P.L., Baron-Cohen S., Tager-Flusberg H., Cohen D. (1993). Pretending and planning. Understanding Other Minds: Perspectives from Autism.

[35] Frith U. (1989). Autism: Explaining the Enigma.

[36] Baron-Cohen S., Leslie A.M., Frith U. (1985). Does the autistic child have a “theory of mind”?. Cognition.

[37] Bigham S.A., Bourchier-Sutton A. (2007). The decontextualization of form and function in the development of pretense. Br. J. Dev. Psychol..

[38] Riviere Á., Rivière A., Martos J. (1998). Tratamiento y definición del espectro autista II: Anticipación, flexibilidad y capacidades simbólicas. El Tratamiento del Autismo. Nuevas Perspectivas.

[39] Mundy P., Sigman M. (1989). The theoretical implications of joint-attention deficits in autism. Dev. Psychopathol..

[40] Jarrold C., Boucher J., Smith P.K. (1996). Generativity defects in pretend play in autism. Br. J. Dev. Psychol..

[41] Lewis V., Boucher J. (1988). Spontaneous, instructed and elicited play in relatively able autistic children. Br. J. Dev. Psychol..

[42] Bentenuto A., De Falco S., Venuti P. (2016). Mother-Child Play: A Comparison of Autism Spectrum Disorder, Down Syndrome, and Typical Development. Front. Psychol..

[43] Mundy P., Sigman M., Ungerer J., Sherman T. (1987). Nonverbal communication and play correlates of language development in autistic children. J. Autism Dev. Disord..

[44] Mundy P., Gomes A. (1998). Individual Differences in Joint Attention Skill Development in the Second Year. Infant. Behav. Dev..

[45] Lewis V., Boucher J., Lupton L., Watson S. (2000). Relationships between Symbolic Play, Functional Play, Verbal and Non-Verbal Ability in Young Children. Int. J. Lang. Commun. Disord..

[46] Spencer P.E. (1996). The association between language and symbolic play at two years: Evidence from deaf toddlers. Child. Dev..

[47] Musatti T., Veneziano E., Mayer S. (1998). Contributions of language to early pretend play. Curr. Psychol. Cogn..

[48] Ricks D.M., Wing L. (1975). Language, communication, and the use of symbols in normal and autistic children. J. Autism Dev. Disord..

[49] Naber F., Bakermans-Kranenburg M.J., Van Ijzendoorn M.H., Swinkels S.H.N., Buitelaar J.K., Dietz C., Van Daalen E., Van Engeland H.M. (2008). Play behavior and attachment in toddlers with autism. J. Autism Dev. Disord..

[50] Rutherford M., Rogers S. (2003). Cognitive Underpinnings of Pretend Play in Autism. J. Autism Dev. Disord..

[51] Lam Y.G., Yeung S.S. (2012). Cognitive deficits and symbolic play in preschoolers with autism. Res. Autism Spectr. Disord..

[52] Warreyn P., Roeyers H., de Groote I. (2005). Early social communicative behaviours of preschoolers with autism spectrum disorder during interaction with their mothers. Autism.

[53] Chang Y., Shih W., Landa R., Kaiser A., Kasari C. (2018). Symbolic Play in School-Aged Minimally Verbal Children with Autism Spectrum Disorder. J. Autism Dev. Disord..

[54] Jarrold C., Boucher J., Smith P.K. (1993). Symbolic play in autism: A review. J. Autism Dev. Disord..

[55] Lee G., Xu S., Guo S., Gilic L., Pu Y., Xu J. (2019). Teaching “Imaginary Objects” Symbolic Play to Young Children with Autism. J. Autism Dev. Disord..

[56] Lee G.T., Feng H., Xu S., Jin S. (2019). Increasing “Object-Substitution” Symbolic Play in Young Children With Autism Spectrum Disorders. Behav. Modif..

[57] Moher D., Liberati A., Tetzlaff J., Altman D.G. (2009). The PRISMA Group. Preferred Reporting Items for Systematic Reviews and Meta-Analyses: The PRISMA Statement. PLoS Med..

[58] Artigas-Pallares J., Paula I. (2012). El autismo 70 años después de Leo Kanner y Hans Asperger. Rev. Asoc. Española Neuropsiquiatr..

[59] Urrútia G., Bonfill X. (2009). Declaración PRISMA: Una propuesta para mejorar la publicación de revisiones sistemáticas y metaanálisis. Med. Clin..

[60] Ogrinc G., Davies L., Goodman D., Batalden P., Davidoff F., Stevens D. (2015). SQUIRE 2.0 (Standards for Quality Improvement Reporting Excellence): Revised publication guidelines from a detailed consensus process. Am. J. Med. Qual..

[61] Riguet C.B., Taylor N.D., Benaroya S., Klein L.S. (1981). Symbolic play in autistic, Down’s, and normal children of equivalent mental age. J. Autism Dev. Disord..

[62] Stone W.L., Lemanek K.L., Fishel P.T., Fernandez M.C., Altemeier W.A. (1990). Play and imitation skills in the diagnosis of autism in young children. Pediatrics.

[63] Libby S., Powell S., Messer D., Jordan R. (1997). Imitation of Pretend Play Acts by Children with Autism and Down Syndrome. J. Autism Dev. Disord..

[64] Libby S., Powell S., Messer D., Jordan R. (1998). Spontaneous Play in Children with Autism: A Reappraisal. J. Autism Dev. Disord..

[65] Dominguez A., Ziviani J., Rodger S. (2006). Play behaviours and play object preferences of young children with autistic disorder in a clinical play environment. Autism.

[66] Rutherford M., Young G., Hepburn S., Rogers S. (2007). A Longitudinal Study of Pretend Play in Autism. J. Autism Dev. Disord..

[67] Hobson R., Lee A., Hobson J. (2009). Qualities of Symbolic Play Among Children with Autism: A Social-Developmental Perspective. J. Autism Dev. Disord..

[68] Pierce H. (2009). Exploratory, Functional and Symbolic Play Behaviors of Toddlers with Autism Spectrum Disorders. Doctoral Thesis.

[69] Hobson J.A., Hobson R.P., Malik S., Bargiota K., Caló S. (2013). The relation between social engagement and pretend play in autism. Br. J. Dev. Psychol..

[70] Hobson J., Hobson R., Cheung Y., Caló S. (2015). Symbolizing as Interpersonally Grounded Shifts in Meaning: Social Play in Children With and Without Autism. J. Autism Dev. Disord..

[71] Fewell R.R., Rich J.S. (1987). Play Assessment as a Procedure for Examining Cognitive, Communication, and Social Skills in Multihandicapped Children. J. Psychoeduc. Assess..

[72] Lifter K., Gitlin-Weinder K., Sandgrund A., Schafer C. (2000). Linking assessment to intervention for children with developmental disabilities or at-risk for developmental delay: The developmental play assessment (DPA) instrument. Play Diagnosis and Assessment.

[73] Lifter K., Sulzer-Azaroff B., Anderson S., Cowdery G.E. (1993). Teaching Play Activities to Preschool Children with Disabilities. J. Early Interv..

[74] Lewis V., Boucher J. (1997). The Test of Pretend Play.

[75] Stagnitti K. (2007). Child.-Initiated Pretend Play Assessment.

[76] Wetherby A., Prizant B. (2002). Communication and Symbolic Behavior Scales Developmental Profile.

[77] Bornstein M.H., O’Reilly A.W. (1993). The Role of Play in the Development of Thought.

[78] Tamis-LeMonda C.S., Bornstein M.H., Rovee-Collier C.R., Lipsitt L.P. (1996). Variation in children’s exploratory, non symbolic, and symbolic play: An explanatory multidimentional framework. Advanced in Infancy Research.

[79] Kavanaugh R.D., Harris P.L. (1994). Imagining the Outcome of Pretend Transformations. Dev. Psychol..

[80] Dawson G., Munson J., Estes A., Osterling J., McPartland J., Toth K., Carver L., Abbot R. (2002). Neurocognitive Function and Joint Attention Ability in Young Children with Autism Spectrum Disorder versus Developmental Delay. Child. Dev..

[81] Misailidi P. (2002). Affective Expressions during Joint Attention Interactions with an Adult: The Case of Autism. Hell. J. Psychol..

[82] Stone W.L., Ousley O.Y., Yoder P.J., Hogan K.L., Hepburn S.L. (1997). Nonverbal Communication in Two and Three Year-Old Children with Autism. J. Autism Dev. Disord..

[83] Jarrold C., Smith P., Boucher J., Harris P. (1994). Comprehension of pretense in children with autism. J. Autism Dev. Disord..

[84] Sigman M., Ruskin E., Arbeile S., Corona R., Dissanayake C., Espinosa M., Kim N., López A., Zierhut C. (1999). Continuity and change in the social competence of children with autism, Down syndrome, and developmental delays. Monogr. Soc. Res. Child. Dev..

[85] Laakso M., Poikkeus A., Eklund K., Lyytinen P. (1999). Social interactional behaviors and symbolic play competence as predictors of language development and their associations with maternal attention-directing strategies. Infant. Behav. Dev..

